# Lung Function After Stereotactic Body Radiation Therapy for Early-Stage Non-Small Cell Lung Cancer, Changes and Predictive Markers

**DOI:** 10.3389/fonc.2021.674731

**Published:** 2021-05-24

**Authors:** Janna Berg, Christina Ramberg, Jon Olav Sulheim Haugstvedt, May-Bente Bengtson, Anne-Marie Gabrielsen, Odd Terje Brustugun, Ann Rita Halvorsen, Åslaug Helland

**Affiliations:** ^1^ Department of Medicine, Vestfold Hospital Trust, Tønsberg, Norway; ^2^ Department of Cancer Genetics, Institute for Cancer Research, Norwegian Radium Hospital, Oslo University Hospital, Oslo, Norway; ^3^ Department of Medical Physics, Oslo University Hospital, Oslo, Norway; ^4^ Department of Radiology and Nuclear Medicine, Vestfold Hospital Trust, Tønsberg, Norway; ^5^ Section of Oncology, Vestre Viken Hospital Trust, Drammen, Norway; ^6^ Department of Oncology, Oslo University Hospital, Oslo, Norway; ^7^ Department of Clinical Medicine, University of Oslo, Oslo, Norway

**Keywords:** non-small cell lung cancer (NSCLC), stereotactic body radiation therapy (SBRT), radiation pneumonitis, pulmonary function test, radiotherapy dose-volume, toxicity

## Abstract

**Introduction:**

The present study explores changes in pulmonary function, symptoms and radiological signs of pneumonitis after curatively intended stereotactic body radiation therapy (SBRT).

**Methods:**

All inoperable, early-stage non-small cell lung cancer patients treated with stereotactic body radiation therapy (SBRT) from 2014-2017 were included in this single-centre study. They were followed regularly for 12 months after treatment. The patients were classified into three groups based on radiology and symptomatology: no radiation pneumonitis, asymptomatic and symptomatic radiation pneumonitis.

**Results:**

Forty-four patients with stage IA-IIB disease were treated with 45–56 Gy in 3–8 fractions. The median age was 75 years, 43% of the patients were female; 60% of the patients had a COPD in GOLD grade of 2-4, and 95.5% were active or former smokers. Symptomatic radiation pneumonitis occurred in 18% of the patients and asymptomatic pneumonitis as defined by radiology, in 39%. The mean of forced expiratory volume in 1 second (FEV1) and diffusion capacity for carbon monoxide (DLCO) decreases for all patients during the first years were higher than one would expect from physiologic ageing. FEV1 and DLCO in percent decrease 7-8% at 1-1.5 months in the symptomatic radiation pneumonitis group. CT scan findings consistent with radiation pneumonitis occurred after a median of 2.9 months in the symptomatic and 5.4 months in the asymptomatic radiation pneumonitis groups. In the group with symptomatic radiation pneumonitis, symptoms, as measured by the Clinical COPD questionnaire score, significantly increased at 3 and 6 months. Significant higher maximum doses to the critical lung volumes DC1000cm^3^ (1000 cm^3^ of lung receiving a given dose or less) and DC 1500cm^3^ (1500 cm^3^ of lung receiving a given dose or less) were observed in patients who developed radiation pneumonitis.

**Conclusion:**

Early decrease in measured FEV1 and DLCO occurred before imaging changes and symptoms and might indicate the development of symptomatic radiation pneumonitis. The dose to critical lung volumes of DC1000 cm^3^ and DC1500 cm^3^ may predict the risk for the development of symptomatic radiation pneumonitis.

## Introduction

Stereotactic body radiation therapy (SBRT) is now the first choice for inoperable, early-stage non-small cell lung cancer (NSCLC) (1, 2). Radiation pneumonitis is a clinically challenging side effect following SBRT for a subset of patients. The reported incidence of radiation pneumonitis varies from 2-4% to 37-47% (3–6). The severity of radiation pneumonitis also varies among different studies; some studies describe only mild radiation pneumonitis, while others report the whole spectrum from mild to severe radiation pneumonitis (3, 7–13). Different grading systems are available; evaluating only symptoms or both symptoms and imaging changes combined (14–17).

Robust predictive markers for radiation pneumonitis are still lacking. Although irradiated volume is known to impact risk, the results are divergent concerning which dosimetric factors and dose-limits have the best predicting powers (5, 6, 18–24). In recent years, radiation dose to critical lung volumes have been introduced to radiotherapy planning and are used to guide planning to avoid too high doses to these volumes, ultimately to avoid lung damage (25, 26). The radiotherapy dose to this specific volume should be below the threshold for the organ to continue functioning for its intended purpose.

Changes in spirometric parameters reported in earlier, mainly retrospective studies, have shown varying results (3–5, 18, 27, 28). Therefore, the aim of this prospective study was to perform a comprehensive and frequent follow-up of pulmonary function as well as identifying predictive markers for the development of radiation pneumonitis with or without symptoms, after curatively intended SBRT.

## Materials and Methods

### Trial Design

This is a prospective, longitudinal, clinical, single-institution (Vestfold Hospital Trust, Tønsberg, Norway) study for patients with early-stage (stage IA-IIB), peripherally located NSCLC (ClinicalTrials.gov NCT02428049).

### Patients

Eligible patients were over 18 years old, with early-stage (stage IA-IIB), peripherally located NSCLC. Tumours were staged in accordance with the Union for International Cancer Control, Tumour, Node, Metastasis staging system version 8 (TNM 8). Patients were technically resectable but deemed medically inoperable by a multidisciplinary tumour board, and the assignment was independent of the study. Patients were included at Vestfold Hospital Trust, Tønsberg, Norway, received SBRT at Oslo University Hospital, Radiumhospitalet, and underwent clinical follow-up at Vestfold Hospital Trust.

### Ethics

All patients provided written informed consent. The study was conducted following legal and regulatory requirements as well as with the general principles outlined in the International Ethical Guidelines for Biomedical Research Involving Human Subjects (Council for International Organizations of Medical Sciences 2002) and the Declaration of Helsinki (World Medical Association 1996 and 2008).

### Assessments and Procedures

#### Radiation Therapy

SBRT was administered as a total dose of 45–56 Gy in 3–8 fractions. The tumour was given an inhomogeneous dose where the prescribed dose encompassed the periphery of the planning target volume (PTV) and the maximum dose in the tumour was about 150% of the prescribed dose. The treatment planning was done on an ordinary CT-series. The respiratory dependent tumour movement was visualised radiologically, and if more than 10 mm, abdominal compression was used to reduce it. This was applied for 9 patients. The dose-volume parameters analysed in this study included the following: the percentages of the lung volume receiving ≥20 and ≥5 Gy (V20-V5), the mean lung dose (MLD) and the total lung volume (subtracted the GTV). Additionally, the dose to the critical lung volume of 1000 and 1500 cm^3^ were determined (DC1000 cm^3^ and DC1500 cm^3^) in line with other publications (26, 29). These parameters were determined as described by Ritter et al. (25). The critical lung volume is the organ volume that will receive a certain radiation dose. For large parallel organs with a lot of redundancy, like the lung or the liver, it is crucial to ascertain that a critical volume of the organs will receive a total dose lower than the threshold dose, in order to avoid end-organ damage. As in the RTOG 1021-study, the recommended maximum dose limits for patients treated with three fractions was 10.5 Gy to 1500 cm^3^ (endpoint pulmonary function) and 11.4 Gy to 1000 cm^3^ (radiation pneumonitis) (25). Three patients were treated for two synchronous tumours in the same lung.

#### Follow-Up Specifications

Follow-up included a physical examination by a pulmonologist, determination of pulmonary function evaluation (**Table 1**) and the Clinical COPD questionnaire at baseline, 1-1,5 months after treatment, and every 3 months thereafter until 12 months after SBRT. CT scans were performed on all follow-up visits except at 1-1,5 months and 9 months when chest X-rays were carried out. Patients with symptoms were also referred for CT scans at 1-1,5 months and 9 months. Data were correlated with radiation dose-volume parameters and clinical and radiological lung toxicity as radiation pneumonitis. After the first year, CT scans of the lung and a physical examination by a pulmonologist, spirometry and determination of the DLCO according to national guidelines were performed two times the second year and yearly for the next three years.

**Table 1:** Baseline characteristics before stereotactic body radiotherapy (SBRT).

Characteristics	Overall, n = 44	No radiation pneumonitis, n = 19 (43%)	Asymptomatic radiation pneumonitis, n=17 (39%)	Symptomatic radiation pneumonitis, n=8 (18%)
Number of tumours	47	21	18	8
**Sex**				
Male	25 (56.8%)	11 (58%)	10 (59%)	4 (50%)
Female	19 (43.2%)	8 (42%)	7 (41%)	4 (50%)
**Median age at diagnosis (years, range)**	75 (51-90)	73 (64-90)	77 (68-89)	71 (51-85)
**Smoke history (%)**				
Former smokers	18 (40.9%)	4 (21%)	10 (59%)	4 (50%)
Active smokers	24 (54.5%)	14 (74%)	7 (41%)	3 (38%)
Never smokers	2 (4.5%)	1 (5%)	0	1 (13%)
**COPD GOLD 2017 classification (%)**				
COPD II-IV (moderate, severe, very severe)	25 (56.8 %)	14 (74%)	10 (59%)	3 (38%)
**Stage (%)**				
T1 (=< 30 mm)	25 (56.8 %)	13 (68%)	7 (41%)	5 (62%)
T2 (> 30 mm to 50 mm)	18 (40.9%)	6 (32%)	9 (53%)	3 (38%)
T3 (>50 mm to 70 mm)	1 (2.3%)	0	1 (6%)	0
**Histology (%)**				
Adenocarcinoma	24 (54.5%)			
Squamous cell carcinoma	12 (27.3%)			
NSCLC NOS	1 (2.3%)			
Unknown	7 (15.9%)			
**PET-CT performed (%)**	38 (86.4%)			
**Pre-existing lung disease (%)**				
Interstitial lung disease	5 (11.4%)	1 (5%)	2 (12%)	2 (25%)
Emphysema	19 (43.2)	11 (58%)	6 (35%)	2 (25%)
**SBRT**				
15 Gy x 3	37	17 (46 %)	13 (35 %)	7 (19 %)
10 Gy x 5	6	2 (33 %)	3 (50 %)	1 (17 %)
7 Gy x 8	1		1 (100 %)	
**Baseline pulmonary function mean (range)**				
FVC (litres)	2.86 (1.42-5.24)	2.98 (1.52-5.24)	2.95 (1.51-4.8)	2.36 (1.42-4.09)
FVC (% of predicted)	88.7 % (39.4-131.7)	89.4% (54.1-131)	94.1 % (60-131.7)	75.8% (39.4-106.2)
FEV1 (litres)	1.80 (0.58-3.62)	1.79 (0.58-3.62)	1.95 (0.58-3.42)	1.51 (0.91-2.18)
FEV1 (% of predicted)	70.9 % (24.8-129.9)	67.5 % (28.9-128)	77.7 % (37.4-129.9)	64.5 % (24.8-103.1)
FEV1/FVC	62 (38-99)	58.1 (38-99)	62.7 (39-82)	68 (42.3-93)
Restrictive impairment	2			2
DLCO single breath (mmol/min/kPa)	4.12 (1.2-7.2)	3.84 (1.2-6.76)	4.37 (1.3-7.2)	4.22 (2.47-6.92)
DLCO (% of predicted)	52.4% (15.8-94)	46.7% (15.8-80)	57.8 % (21-87)	53.6 (23.7-94)
DLCO/VA (mmol/min/kPa/litres)	0.84 (0.28-1.5)	0.73 (0.28-1.48)	0.89 (0.35-1.48)	0.98 (0.47-1.5)
DLCO/VA (% of predicted)	64.5% (20.3-119)	56.5 (20.3-115.4)	69.6 % (27.2-119)	71.4 (33.8-106)
TLC (litres)	6.62 (3.24-10.74)	7.32 (3.39-10.74)	6.38 (3.66-10.45)	5.61 (3.24-9.29)
TLC (% of predicted)	109% (47-168)	117.4 (47-168)	107.8 % (65-166)	93.7 (67-125)
RV (litres)	3.96 (1.31-8.88)	4.71 (2.58-8.88)	3.59 (1.31-7.44)	3.26 (1.64-6.99)
RV (% of predicted)	161% (59-312)	189 % (94-312)	145.8 % (59-278)	138.7 (76-290)
ITGV (litres)	4.92 (2.07-9.41)	5.75 (3.53-8.93)	4.65 (2.71-9.41)	3.79 (2.07-7.31)
ITGV (% of predicted)	151% (73-275)	173 % (100-275)	145% (78-252)	118.7 (73-198)
Arterial blood gas				
pO2 (kPa)	10.23 (7.9-12.8)	10.2 (8.2-12)	10.3 (8.4-12.8)	10.2 (7.9-11.9)
pCO2 (kPa)	5.04 (4.18-6.5)	4.90 (4.20 - 6)	5.16 (4.18-6.3)	5.1 (4.4.-6.5)
6MWT				
Walk distance (meters)	436 (280-615)	399 (280-560)	469 (350-615)	472 (365-560)
SaO2 before (%)	96% (90-99)	96% (92-99)	96 (95-98)	94 (90-97)
SaO2 minimum (%)	92% (85-97)	92 (87-96)	93 (88-97)	91 (85-97)
Borg score after (points)	3.47 (0-10)	3.6 (1-7)	3.3 (0-10)	5 (4-6)
CCQ				
Total score (points)	1.32 (0-4.25)	1.55 (0-4.25)	0.96 (0-3.2)	1.50 (0.01-2.9)
Symptoms score (points)	1.67 (0-5)	2.01 (0-5)	1.13 (0-3)	1.94 (0-4.25)
Functions score (points)	1.35 (0-4.5)	1.41 (0-4.5)	1.14 (0-3.5)	1.59 (0-3)
Mental score (points)	0.66 (0-4)	0.82 (0-4)	0.56 (0-3)	0.5 (0-2)

COPD, Chronic obstructive pulmonary disease; CCQ, Clinical COPD questionnaire, DLCO. Diffusion capacity for carbon monoxide; FEV1, Forced expiratory volume in one second; FVC, Forced vital capacity; GOLD, Global Initiative for Chronic Obstructive Lung Disease; ITGV, Intrathoracic gas volume; NSCLC, Non-small cell lung cancer; NOS, not otherwise specified; PaCO2, Partial pressure of carbon dioxide; PaO2, Partial pressure of oxygen; PET-CT, Positron emission tomography - computer tomography; RV, Residual volume; SaO2, Oxygen saturation; TLC, Total lung capacity.

#### Grading of Radiation Pneumonitis

The patients’ symptoms were graded according to The Common Terminology Criteria for Adverse Events (CTCAE). Radiological changes were graded according to the European Organisation for Research and Treatment of Cancer and Late Effects Normal Tissues-Subjective, Objective, Management, Analytic (EORTC/LENT-SOMA). Based on the CTCAE and EORTC/LENT-SOMA grading, the patients were divided into 3 following groups:

1. The no radiation pneumonitis group: Patients with mild symptoms equivalent CTCAE grade 0-1 and with no or only slight changes on radiology equivalent EORTC (LENT-SOMA) grade 0-1.
2. The asymptomatic radiation pneumonitis group: Patients with mild symptoms equivalent CTCAE grade 0-1 and with patchy or increased density on imaging equivalent EORTC (LENT-SOMA) grade 2-3.
3. The symptomatic radiation pneumonitis group: Patients with symptoms equivalent to CTCAE grade 2-5 and with patchy or increased density on imaging equivalent EORTC (LENT-SOMA) grade 2-3. CTCAE grade 2 represents the need for some medical intervention (e.g., steroids), and grade 3 indicates the use of supplemental oxygen (14).

All CT scans were evaluated by an experienced thoracic radiologist focusing on radiation pneumonitis.

#### Pulmonary Function Evaluation

Spirometry, gas diffusion capacity and static lung volume measurements were performed according to the American Thoracic Society (ATS)/European Respiratory Society (ERS) guidelines (30).

COPD was diagnosed according to the criteria of the Global Initiative for Chronic Obstructive Lung Disease (GOLD). Body plethysmography (TLC, RV and ITGV) provides additional information on COPD and in restrictive disorders (30).

#### The Clinical COPD Questionnaire

The Clinical COPD questionnaire was developed as a simple tool to help clinicians identify both the clinical status of the airways, activity limitations and emotional dysfunction in COPD patients (31). The Clinical COPD questionnaire is a validated patient-reported outcome tool to assess functional performance (31, 32). The Clinical COPD questionnaire was appropriate for this study because the aim of this study is research on radiation pneumonitis, which is dominated by respiratory, COPD-like symptoms, and most of the study patients had COPD.

### Statistical Methods

Data are reported using descriptive statistics with percentages, means, medians and ranges. The results are given as the mean values with 95% confidence intervals (CIs) unless otherwise stated. STATA version 15.1 (Stata/MP, StataCorp Texas, USA) was used to perform pulmonary function test calculations and produce graphical figures. Changes in pulmonary function test results were measured using a linear mixed model for repeated measurements with a subject-specific random intercept and maximum likelihood estimation. To explore the relationship between dose-volume parameters and lung toxicity, Spearman’s rank correlation was utilized (R version 3.6.2). Plots were created using the ggplot2 package in R Wickham (33). Kruskal-Wallis tests were used to evaluate significant differences in the radiotherapy dose-volume among the radiation pneumonitis groups (R version 3.6.2). Fisher’s exact test was used to compare between the study populations at baseline. Two-sided P-values <0.05 were considered statistically significant.

## Results

### Patients

**Figure 1:**
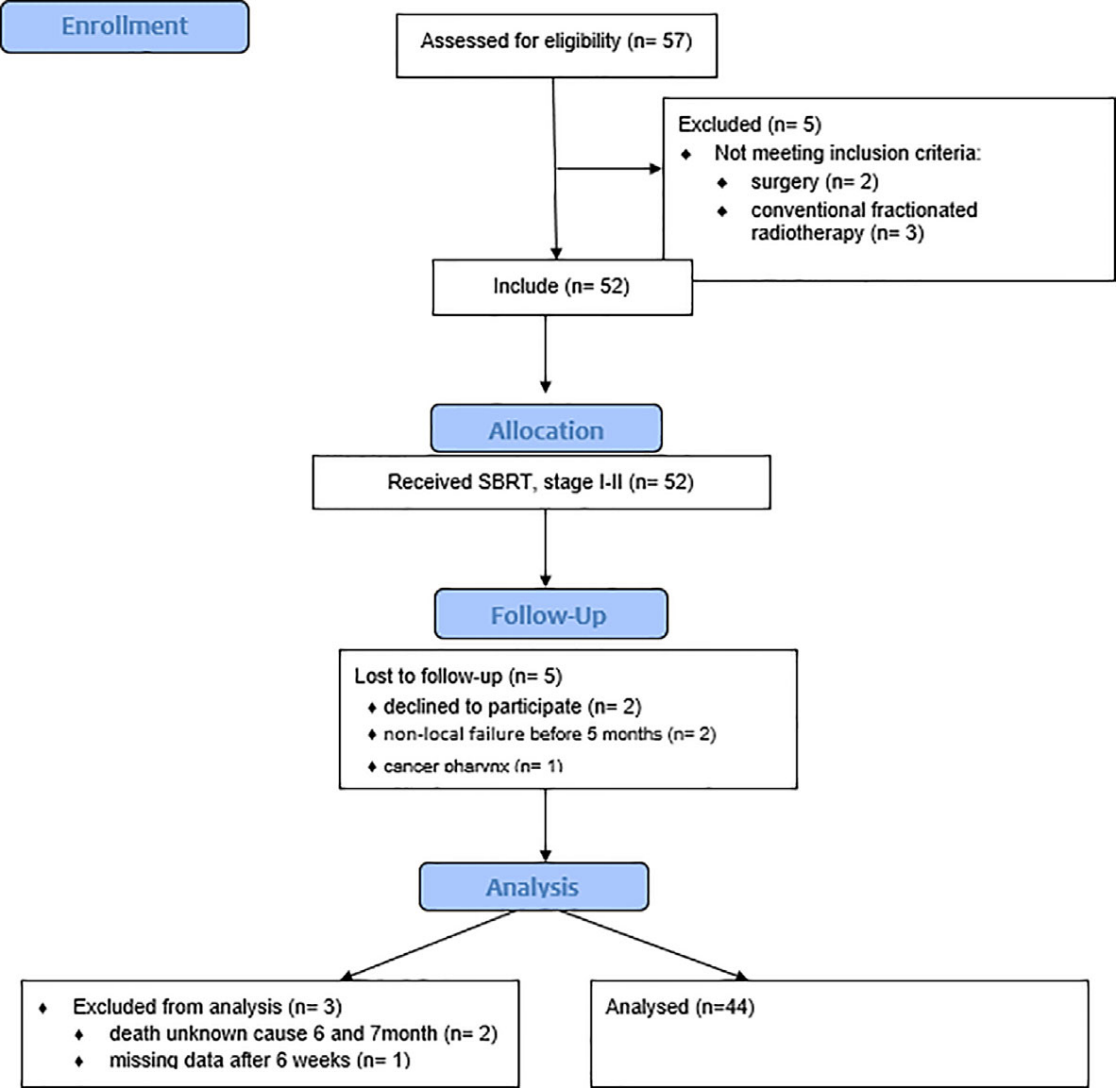
The study flow diagram.

The study population and baseline characteristics of the patients in this study are presented in **Table 1**. The CONSORT study flow diagram is presented in **Figure 1**. From February 2014 until December 2017, 57 patients were assessed for eligibility and 44 were included in this study. Five (11,4%) of the 44 patients were diagnosed with non-local failure within the first year of follow-up.

### Lung Toxicity

Detailed patients’ characteristics are presented in **Table 1**. Of a total of eight patients in the symptomatic radiation pneumonitis group, five experienced grade 2 toxicity and tree grade 4 toxicity.

Radiation pneumonitis on CT occurred after a median of 4.9 months for all patients, 5.1 months for asymptomatic and 2.9 months for symptomatic radiation pneumonitis patients.

Active smokers were significantly less susceptible to the development of radiation pneumonitis (p-value=0.04) according to Fisher’s exact test. Patients with emphysema showed a trend towards being less susceptible (p-value=0.09) to developing radiation pneumonitis.

### Changes in Pulmonary Function Tests Following SBRT, All Patients

Comprehensive baseline tests results are presented in **Table 1**. Results of comprehensive tests distributed across the timeline are presented in Supplementary table 1.

The mean drop in FEV1 for all patients, was 0,17 litre and DLCO 0.35 mmol/min/kPa (1.1 ml/min/mm Hg) at one year.

A linear mixed model analysis at the different assessment times showed significant differences from the mean baseline values in percent of predicted for FVC, FEV1, DLCO and in the six-minute walking test (**Table 2**). No significant changes in the TLC, RV or ITGV were observed.

**Table 2:** Significant delta percent changes in mean values from baseline according to a linear mixed model.

Pulmonory function test	Time in months post-SBRT	All patients (n=44)		No radiation pneumonitis (n=19)		Asymptomatic radiation pneumonitis (n=17)		Symptomatic radiation pneumonitis (n=8)	
		**% delta from baseline [CI]**	**p-value**	**% delta from baseline [CI]**	**p-value**	**% delta from baseline [CI]**	**p-value**	**% delta from baseline [CI]**	**p-value**
FVC % of predicted	3	n.s.	n.s.	n.s.	n.s.	n.s.	n.s.	- 9.2 [-17.5;-0.8]	0.03
	6	n.s.	n.s.	n.s.	n.s.	- 5.6 [-10.6;-0.5]	0.03	n.s.	n.s.
	9	n.s.	n.s.	n.s.	n.s.	n.s.	n.s.	n.s.	n.s.
	12	- 4.0 [-7.6;-0.3]	0.03	n.s.	n.s.	- 7.4 [12.6; -2.2]	0.01	n.s.	n.s.
FEV1 % of predicted	3	n.s.	n.s.	n.s.	n.s.	n.s.	n.s.	- 10.2 [-17.0; -3.4]	<0.01
	6	- 4.1 [-6.9; -1.3]	<0.01	n.s.	n.s.	- 6.0 [-10.7; -1.2]	0.01	- 9.0 [-15.8;-2.2]	0.01
	9	- 4.1 [-7.0; -1.2]	<0.01	n.s.	n.s.	n.s.	n.s.	- 9.9 [-17.3;-2.5]	0.01
	12	- 4.6 [-7.4;-1.7]	<0.01	n.s.	n.s.	n.s.	n.s.	- 10.9 [-18.0; -3.8]	<0.01
DLCO % of predicted	3	-3.6 [-6.4;-0.8]	0.01	n.s.	n.s.	n.s.	n.s.	- 9.0 [-16.5;-1.4]	0.02
	6	- 3.6 [-6.5;-0.8]	0.01	n.s.	n.s.	n.s.	n.s.	- 8.3 [-15.9;-0.8]	0.03
	9	-4.0 [-7.0; 0.9]	0.01	n.s.	n.s.	n.s.	n.s.	n.s.	n.s.
	12	- 3.9 [-6.9; -1.0]	<0.01	n.s.	n.s.	n.s.	n.s.	- 11.0 [-18.8;-3.1]	<0.01
Blood gas, pO2 (kPa)	6	n.s.	n.s.	+ 0.9 [0.1; 1.7]	0.02	n.s.	n.s.	n.s.	n.s.
Blood gas, pCO2 (kPa)	9	n.s.	n.s.	n.s.	n.s.	n.s.	n.s.	-0.7 [-1.1; -0.3]	<0.01
CCQ total (points)	3	n.s.	n.s.	n.s.	n.s.	n.s.	n.s.	+ 0.8 [0.1;1.6]	0.04
	6	n.s.	n.s.	n.s.	n.s.	n.s.	n.s.	+ 0.8 [0.1; 1.5]	0.03
6MWT, walk distance (meters)	3	-31.7 [-62.9; -0.6]	0.046						
	6	- 47.8 [-79.8; -15.9]	<0.01	n.s.	n.s.	n.s.	n.s.	n.s.	n.s.
	9	- 41.0 [-74.7; -7.3]	0.02	n.s.	n.s.	n.s.	n.s.	n.s.	n.s.
6MWT, SaO2 min (%)	6	- 4.0 [-6.8; -1.2]	<0.01	n.s.	n.s.	n.s.	n.s.	n.s.	n.s.
									

FVC, Forced vital capacity; FEV1, Forced expiratory volume the first second; DLCO, Diffusing capacity for carbon monoxide; pO2, Partial pressure of oxygen; pCO2, Partial pressure of carbon dioxide; CCQ, The Clinical COPD Questionnaire; n.s, Not significant; 6MWT, Six-minute walking test; SaO2, Blood-oxygen saturation; SBRT, stereotactic body radiation therapy.

### Changes in Pulmonary Function Tests and the Clinical COPD Questionnaire Results Following SBRT in Three Groups: No, Asymptomatic, and Symptomatic Radiation Pneumonitis

Comprehensive baseline tests results are presented in **Table 1**.

**Figure 2:**
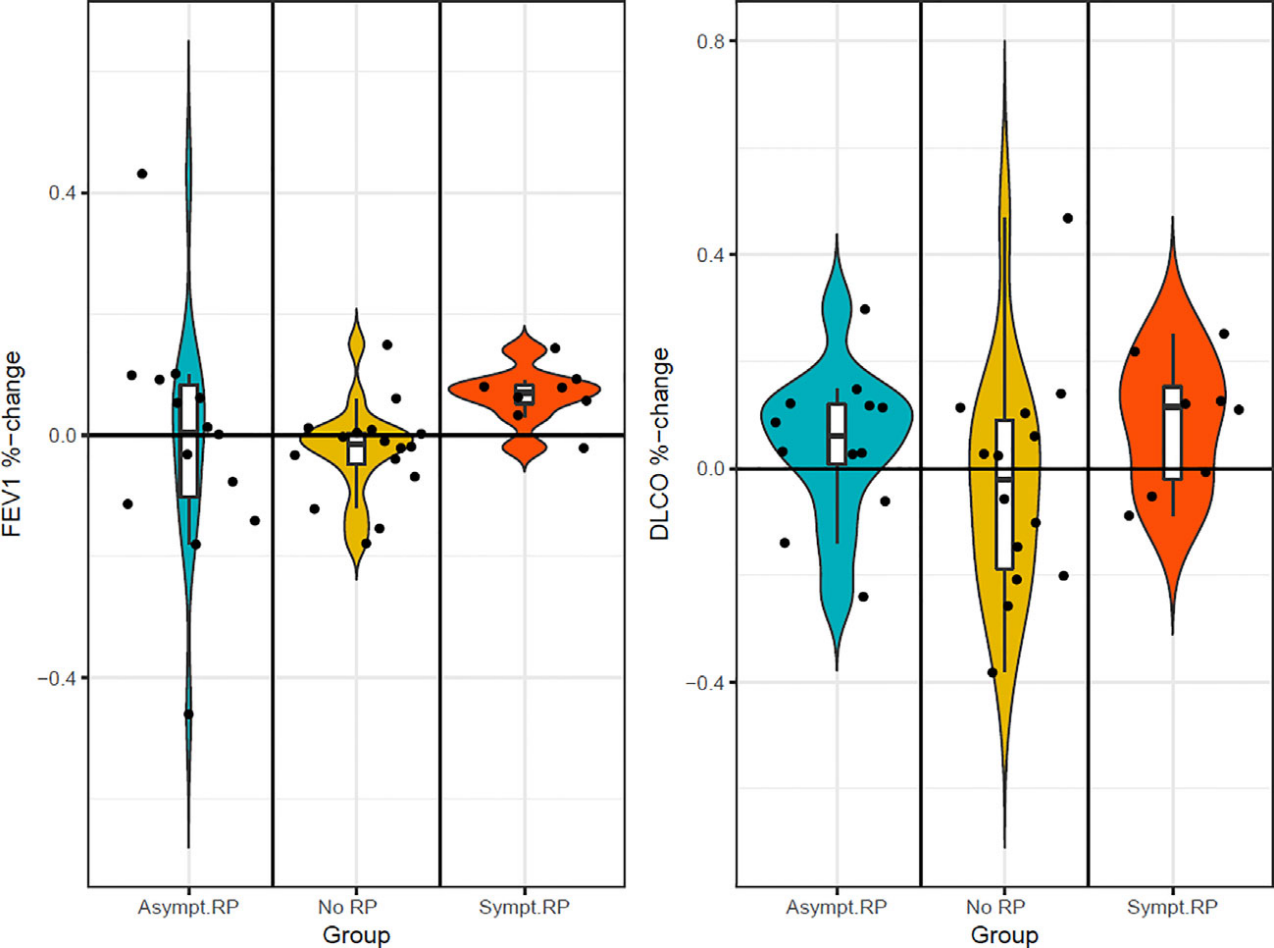
FEV1 and DLCO in percent changes in three groups at 1– 1.5 months.

**Figure 3:**
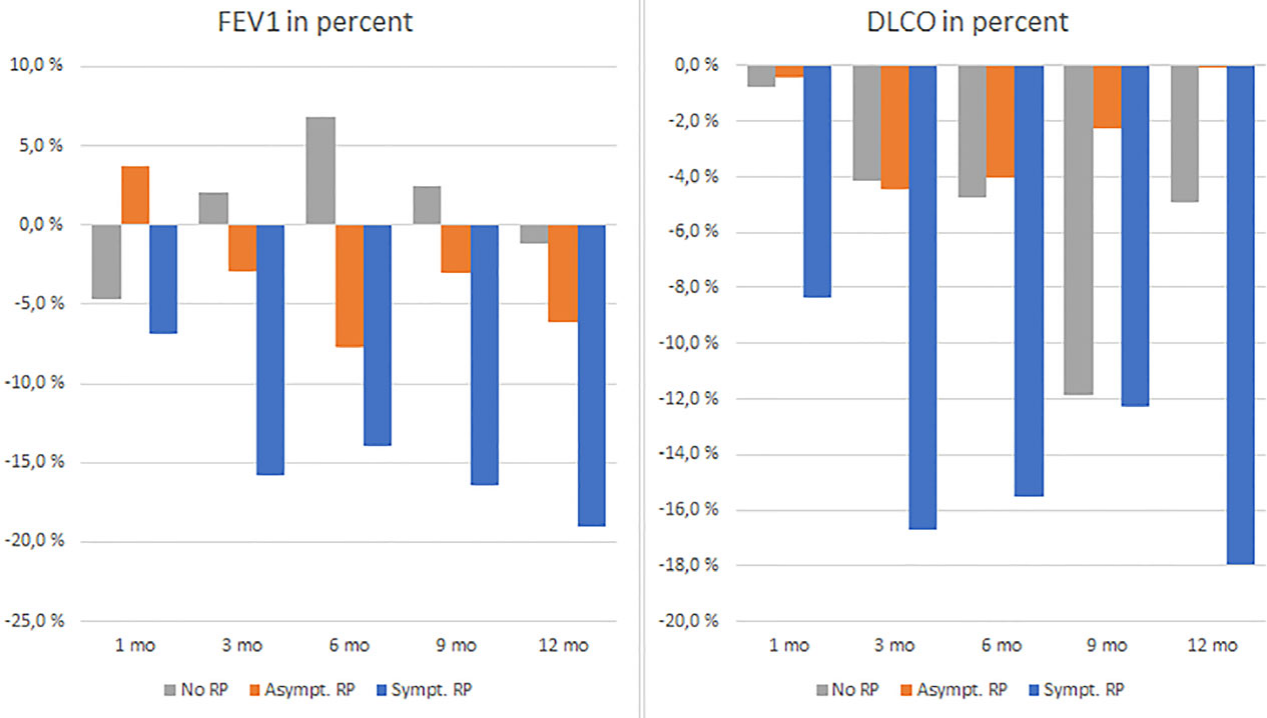
FEV1 and DLCO in percent changes in three groups 1–12 months.

In patients with symptomatic radiation pneumonitis, the DLCO and FEV1 in percent had 7-8% drop at 1-1,5 months after SBRT (**Figure 2**. DLCO and FEV1 in percent changes at 1-1.5 months and **Figure 3**. DLCO and FEV1 in percent changes for one year), much earlier than radiologic changes which occurred after a median of 3 months. Symptoms measured by the Clinical COPD questionnaire total score was significantly increased at 3 and 6 months in a linear mixed model.

According to a linear mixed model, significant changes in several tests results, i.a. FEV1 and DLCO in percent were observed at most of the assessment times in the symptomatic radiation pneumonitis group. A comprehensive overview is given in **Table 2**.

**Figure 4:**
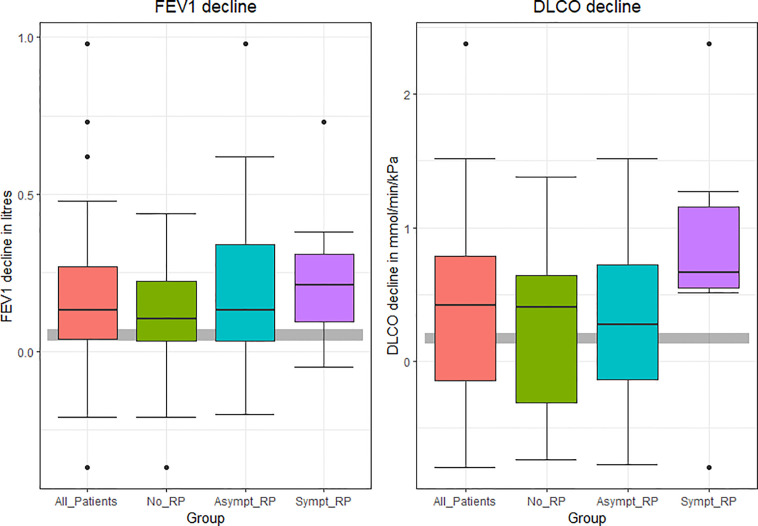
FEV1and DLCO decline in all patients and in three groups after 12 months. The gray stripe is expected physiological changes.

The mean drops in absolute values in FEV1 and DLCO after 12 months are presented in **Figure 4** (FEV1 and DLCO decline in all patients and in three groups after 12 months).

### Correlation Between Dose-Volume Parameters, Lung Toxicity, and Changes in Pulmonary Function

**Figure 5:**
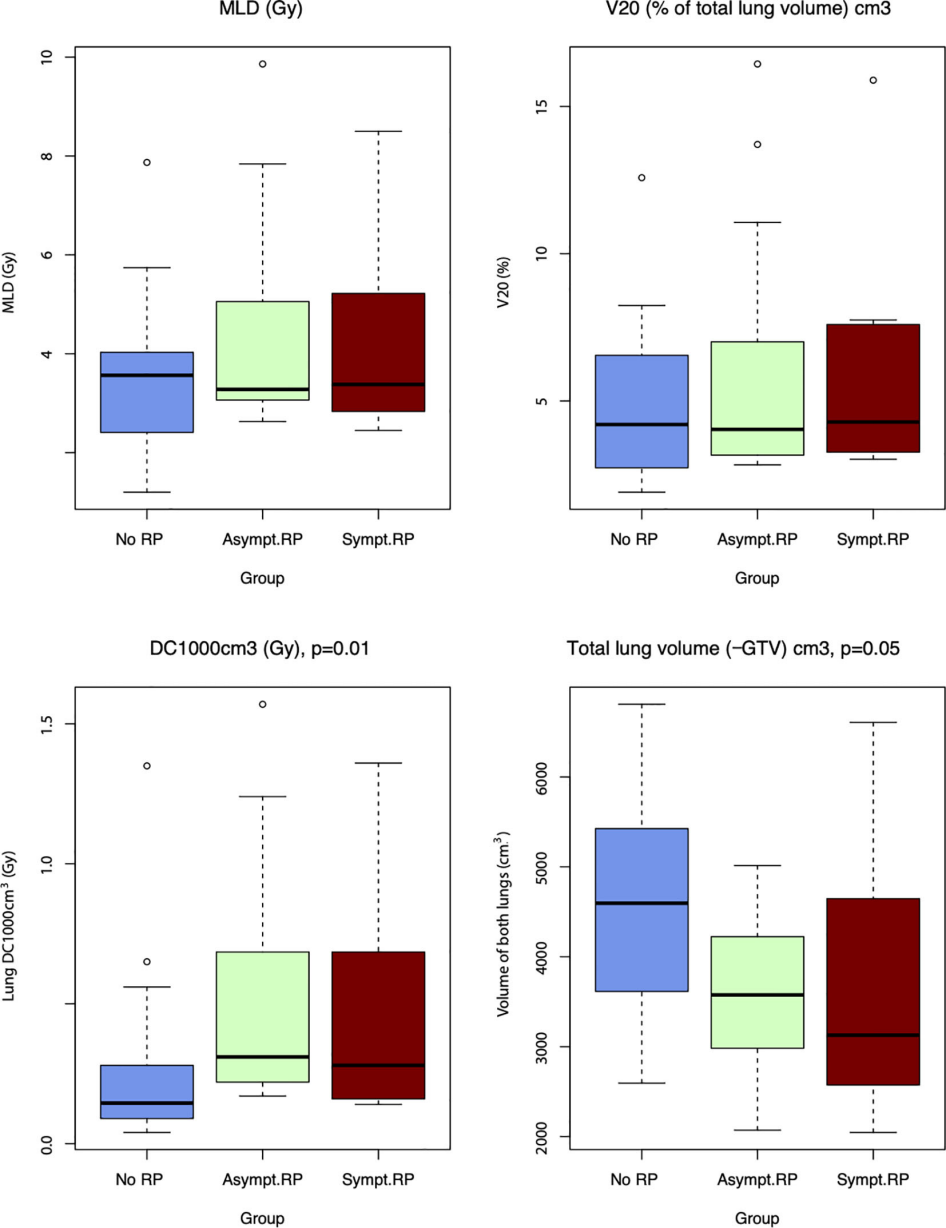
Correlation between MLD, V20, DC1000 cm3, Total lung volume cm^3^ and lung toxicity.

When comparing the three groups, the maximum dose to the critical lung volumes, DC1000 cm^3^ (p-value = 0.010) and DC1500 cm^3^ (p-value = 0.017), was significantly associated with the development of radiation pneumonitis (**Figure 5**. Correlation between MLD, V20, DC1000 cm^3^, Total lung volume cm^3^ and lung toxicity). However, the DC1000 cm^3^ and DC1500 cm^3^ doses were substantially less than the recommended threshold dose in clinical use.

The total lung volume (subtracted the GTV) was lower in the radiation pneumonitis group than in the no radiation pneumonitis group, with borderline significance (p=0.054). The other dose-volume parameters were not significantly associated with the development of radiation pneumonitis.

On Spearman’s rank correlation, several dosimetric parameters were significantly negatively correlated with a drop in the FEV1 in the asymptomatic and symptomatic radiation pneumonitis groups but not in the no radiation pneumonitis group (**Table 3**). Significant correlations already occurred at one month for the symptomatic radiation pneumonitis group, while the asymptomatic group first showed significant correlations at six months.

**Table 3:** Significant negative correlations between dosimetric parameters and FEV1-drop (%) according to Spearman correlation.

	At 1 month			At 6 months		
Dosimetric parameters	No radiation pneumonitis	Asymptomatic radiation pneumonitis	Symptomatic radiation pneumonitis	No radiation pneumonitis	Asymptomatic radiation pneumonitis	Symptomatic radiation pneumonitis
MLD (Gy)	n.s.	n.s.	-0.83 (p=0.01)	n.s.	-0.75 (p<0.01)	n.s.
V5Gy (cm3) (% of total lung volume)	n.s.	n.s.	-0.81 (p=0.01)	n.s.	-0.72 (p<0.01)	n.s.
V20Gy (cm3) (% of total lung volume)	n.s.	n.s.	-0.79 (p=0.02)	n.s.	-0.75 (p<0.01)	n.s.
DC1000ccm (Gy)	n.s.	n.s.	-0.79 (p=0.02)	n.s.	-0.7 (p<0.01)	n.s.
GTV (cm3)	n.s.	n.s.	-0.72 (p=0.04)	n.s.	-0.57 (p=0.02)	n.s.

GTV, Gross tumour volume; DC1000cm3/1500cm3, Dose to the critical lung volume (subtracted the GTV); MLD, Mean lung dose; n.s, Not significant; RP , Radiation pneumonitis; V5, Percentages of lung volumes receiving ≥5 Gy (subtracted the GTV); V20 Percentages of lung volumes receiving ≥20 Gy (subtracted the GTV).

## Discussion

### Predictive Markers for Radiation Pneumonitis

We demonstrated that a drop in the DLCO and FEV1 occurring 1-1.5 months after SBRT is associated with the development of symptomatic radiation pneumonitis, while symptoms and imaging changes occurred later than 3 months for most patients. This finding is supported by a strong association between several dosimetric parameters and a drop in the FEV1 at 1-1,5 months in the same group. Interestingly, the asymptomatic radiation pneumonitis group showed the same association between FEV1 and dosimetric parameters at 6 months. As expected, changes in FEV1 did not correlate with any dosimetric parameters in patients without radiation pneumonitis. Trials with larger sample sizes are required to verify the results.

We found that another possible predictive marker for radiation pneumonitis is an early (2.8 months) increase in density on CT scans, which has also been found in other studies (19, 20, 34). Early radiographic changes might indicate vulnerable lung tissue.

In the present study, we demonstrated that active smokers and patients with emphysema presented less radiation pneumonitis, which is in line with earlier studies (6, 13, 35). The reduced tissue density of the terminal bronchioles and destroyed alveolar walls in COPD probably cause less tissue inflammation. Smoking can also lead to reduced radiation-induced inflammation (36).

### Changes in Pulmonary Function Tests and the Clinical COPD Questionnaire Results Following SBRT

In this study, we demonstrated significant decreases in the percent FVC, FEV1 and DLCO after SBRT in all patients, which is in line with two previous studies (4, 5). Some studies (3, 18, 27, 28) found no significant changes in the FVC and FEV1 after SBRT, and some reported only a decrease in the DLCO (4, 18, 28). Differences in the patient cohorts, including the occurrence of COPD and the severity or definition of radiation pneumonitis, could explain the varying results of the above studies.

Mean decreases in the absolute values for all patients in FEV1 and DLCO (due to symptomatic radiation pneumonitis) during the first year after SBRT, was higher in our study than one would expect from physiologic ageing (37–41).

### Grading Systems for the Evolution of Radiation Pneumonitis

Various radiation pneumonitis incidences in different studies (2-47%) are probably caused by the use of different classification systems, study populations and different radiation pneumonitis grade interpretations. Our study supports the importance of imaging assessments in addition to symptoms in evaluating radiation pneumonitis. Pulmonary symptoms of radiation pneumonitis can also be typical for other diseases, such as COPD exacerbation, pneumonia, lung embolism, heart insufficiency, pre-existing lung cancer and cancer recurrence.

Dividing patients into three groups (no radiation pneumonitis, asymptomatic and symptomatic radiation pneumonitis) led to the important recognition of some clear features for the early diagnosis of at-risk patients.

### Impact of the Dose-Volume and the Total Lung Volume on Radiation Pneumonitis After SBRT in NSCLC

The development of radiation pneumonitis after SBRT in this study was associated with a higher maximum dose to the critical lung volumes of DC1000 cm^3^ and DC1500 cm^3^ and a lower total lung volume (subtracted the GTV). However, the DC1000 cm^3^ and DC1500 cm^3^ values were well within the recommended limits, which might indicate a need for refining these recommended thresholds. This information will probably be important in calculations for further refinement of the critical volume of an individual organ. First, females usually have lower total lung volume than males. Second, the calculated one-third residual postradiotherapy volume, the critical volume, can be too small to maintain function and cause a predisposition for radiation pneumonitis in patients with a partially reduced lung volume after previous surgeries or radiation therapy. In agreement with our study, other studies (23, 42) have suggested that further research should be undertaken for verification. Minimising the irradiated volume is important for reducing the risk for development of pneumonitis. In this study, abdominal compression was used to reduce the respiratory movement during irradiation.

### Study Limitations

The present study has several limitations. This was a single-institution study with a limited number of patients. The follow-up period was relatively short, at one year. The radiation pneumonitis grading process was not fully blinded to patient identity even though the radiologist graded pneumonitis in a blinded manner. The various radiation doses used in this study are another limitation; however, a statistical examination of the different groups showed that radiation pneumonitis was not more likely to develop for any particular fractionation scheme.

## Conclusion

Early decrease in FEV1 and DLCO, occurring before imaging changes and symptoms, can indicate the development of symptomatic radiation pneumonitis. A CT scan within 3 months should be considered in this case. Patients with a small lung volume (e.g., women and patients with a history of lung surgery or radiotherapy) and never-smokers are more prone to developing radiation pneumonitis, unlike patients with emphysema. Radiation dose to critical lung volumes (DC1000 cm^3^ and DC1500 cm^3^) are relatively new dose parameters and are important factors to consider when planning SBRT of the lung that need more research.

## Data Availability Statement

The original contributions presented in the study are included in the article/**Supplementary Material**. Further inquiries can be directed to the corresponding author.

## Ethics Statement

The studies involving human participants were reviewed and approved by Regional Committees for Medical Research Ethics - South East Norway, ref.2013/169. The patients/participants provided their written informed consent to participate in this study.

## Author Contributions

Conception and design: JB, OTB, ARH, ÅH. Provision of study materials or patients: JB, MBB, ÅH. Collection and assembly of data: JB, CR, JOSH, ÅH. Data analysis and interpretation: JB, CR, MBB, AMG, OTB, ARH, ÅH. Manuscript writing: JB, CR, ARH, ÅH. All authors contributed to the article and approved the submitted version.

## Funding

This research was funded by the Regional Health Authorities in Southeast Norway (grant 2015058), the Vestfold Hospital Trust (grant 197430) and an unrestricted grant from Boehringer Ingelheim Norway (grant 197430).

## Conflict of Interest

The authors declare that the research was conducted in the absence of any commercial or financial relationships that could be construed as a potential conflict of interest.

## Supplementary Material

Supplementary Table 1 Supplement to Materials and Methods.

Supplementary Table 2 Results of comprehensive tests distributed across the timeline, all patients.

Supplementary Table 3 Study data.

## Abbreviations

COPD, Chronic obstructive pulmonary disease; CTV, Clinical target volume; DC1000 cm^3^/1500 cm^3^, Dose to the critical lung volume (subtracted the GTV); DLCO, Diffusion capacity for carbon monoxide; FEV1, Forced expiratory volume in one second; FVC, Forced vital capacity; GTV, Gross tumour volume; ITGV, Intrathoracic gas volume; MLD, Mean lung dose; PaCO2 Partial pressure of carbon dioxide; PaO2, Partial pressure of oxygen; PTV, Planning target volume; RV, Residual volume; SBRT Stereotactic body radiation therapy; TLC, Total lung capacity.
