# Supplementary material for: Lung Function After Stereotactic Body Radiation Therapy for Early-Stage Non-Small Cell Lung Cancer, Changes and Predictive Markers
Source: Front Oncol. 2021 May 24;11:674731. doi: 10.3389/fonc.2021.674731 (PMC8181743; doi:10.3389/fonc.2021.674731)
Supplement: Supplementary Table 1 — Supplement to Materials and Methods. [file Table_1.docx]

Supplementary Material

# Materials and Methods

## Assessments and procedures

### Radiation therapy

Delineation of the gross tumour volume (GTV) was done on a maximum intensity projection (MIP) CT-series, and then expanded by 5 mm to the clinical target volume (CTV). The PTV included the CTV plus an additional 5 mm margin uniformly applied to the CTV. All patients were treated with arc therapy, static or volumetric-modulated arc therapy (VMAT) on either a Varian TrueBeam STx linear accelerator (Varian Medical Systems, Inc., USA) or an Elekta Synergy linear accelerator (Elekta AB, Sweden). Cone-beam CT image guidance was carried out prior to each treatment. Standard international limits for organs at risk were evaluated by oncologists and physicists.

### Follow-up specifications

Follow-up included a physical examination by a pulmonologist, spirometry, the diffusion capacity for carbon monoxide (DLCO), determination of the total lung capacity (TLC), residual volume (RV) and intrathoracic gas volume (ITGV) by body plethysmography, determination of the partial pressure of carbon dioxide (PaCO2) and oxygen (PaO2) by arterial blood gas analysis, the six-minute walking test, the Clinical COPD questionnaire and blood sample analysis at baseline, 1-1,5 months after treatment, and every 3 months thereafter until 12 months after SBRT.

### Arterial blood gas

Arterial blood for the analysis of PaO2 and PaCO2 in kilo Pascal (kPa) was drawn after 5 minutes of rest, breathing room air. For analyses, we used a Radiometer ABL 735 (Radiometer, Copenhagen, Denmark).

### Six-minute walking test

The six-minute walking test was performed according to the ATS/ERS guidelines with a walking course of 35 m in length (1). Although the six-minute walking test assesses the submaximal level of functional capacity because patients choose their own intensity of exercise, this may better reflect the level for daily physical activities and is the most reliable semi-laboratory functional capacity test.

### Pulmonary function evaluation

Spirometry, gas diffusion capacity and static lung volume measurements were performed according to the American Thoracic Society (ATS)/European Respiratory Society (ERS) guidelines (2). The pulmonary function tests were performed using the Jaeger Master Lab device (Eric Jaeger, Wurzburg, Germany). The recorded variables were the forced expiratory volume in 1 second (FEV1), forced vital capacity (FVC), FEV1/FVC, DLCO, DLCO divided by the alveolar lung volume (DLCO/VA), TLC, ITGV, and RV, as well as all of those measurements in terms of the percent of the predicted value.



COPD was diagnosed according to the criteria of the Global Initiative for Chronic Obstructive Lung Disease (GOLD). Body plethysmography (TLC, RV and ITGV) provides additional information on COPD and in restrictive disorders (30).

# Supplementary Figures and Tables

For more information on Supplementary Material and for details on the different file types accepted, please see [here](http://home.frontiersin.org/about/author-guidelines#SupplementaryMaterial). Figures, tables, and images will be published under a Creative Commons CC-BY licence and permission must be obtained for use of copyrighted material from other sources (including re-published/adapted/modified/partial figures and images from the internet). It is the responsibility of the authors to acquire the licenses, to follow any citation instructions requested by third-party rights holders, and cover any supplementary charges.

**References**

1. Singh SJ, Puhan MA, Andrianopoulos V, Hernandes NA, Mitchell KE, Hill CJ, et al. An official systematic review of the European Respiratory Society/American Thoracic Society: measurement properties of field walking tests in chronic respiratory disease. Eur Respir J. 2014;44(6):1447-78.

2. Brusasco V, Crapo R, Viegi G, American Thoracic S, European Respiratory S. Coming together: the ATS/ERS consensus on clinical pulmonary function testing. Eur Respir J. 2005;26(1):1-2.
